# Revisiting Thermal Gradient Experiments: Effects of Thermal Heterogeneity on Salamander Behavior

**DOI:** 10.1093/iob/obaf015

**Published:** 2025-04-08

**Authors:** J Laterza-Barbosa, R Rainha, A Flores-Guzman, A K Ting, J Chen, E A Riddell, M M Muñoz, C A Navas

**Affiliations:** Department of Ecology and Evolutionary Biology, Yale University, New Haven, CT, 06520, United States; Department of Ecology and Evolutionary Biology, Yale University, New Haven, CT, 06520, United States; Instituto Nacional de Pesquisas da Amazônia (INPA), Manaus, AM, 69.067-375, Brazil; Department of Ecology and Evolutionary Biology, Yale University, New Haven, CT, 06520, United States; Department of Ecology and Evolutionary Biology, Yale University, New Haven, CT, 06520, United States; Department of Ecology and Evolutionary Biology, Yale University, New Haven, CT, 06520, United States; Department of Biology, University of North Carolina at Chapel Hill, Chapel Hill, NC, 27599, United States; Department of Ecology and Evolutionary Biology, Yale University, New Haven, CT, 06520, United States; Department of Ecology and Evolutionary Biology, Yale University, New Haven, CT, 06520, United States; Departamento de Fisiologia, Instituto de Biociências, Universidade de São Paulo, São Paulo, SP, 05508-090

## Abstract

Thermal gradient experiments are commonly used in studies of ectothermic organisms for a variety of scientific inquiries. Such gradient experiments, performed in the laboratory, are often used to infer the climatic preferences of animals in the absence of other variables. However, the ability to extrapolate laboratory results to the field is only as good as the accumulation of ecological data for that organism. When the variable quantified is interpreted as thermal “preference,” there are some assumptions that come with it, namely that the organism selects a particular preferred temperature by positive thermotaxis. Amphibians, as well as most ectotherms, tend to be thermoconformers, so conclusions from thermal gradient experiments carry different meanings than they do for organisms such as heliothermic ectotherms that maintain a narrow range of body temperatures in the lab and field. We tested whether and how the Eastern Red-backed Salamander (*Plethodon cinereus*) behaves when presented with a heterothermal gradient arena in comparison to a control (homothermal) arena. Salamanders in the control arena unambiguously moved toward either end of the arena, despite no variation in temperature being available. We found that salamanders did respond to a thermal gradient, but that their thermoregulatory behavior was limited to the avoidance of the hottest end of the gradient, and not a positive thermotaxis toward a specific temperature as assumed of a thermal “preference.” Our results encourage a broader consideration of how laboratory-measured behaviors relate to the predicted behaviors of organisms in natural settings, and a re-evaluation of the terminology used to describe movement behaviors in thermal gradients.

## Resumen

Los experimentos con gradientes térmicos se utilizan comúnmente en estudios de organismos ectotermos para una variedad de preguntas científicas. Dichos experimentos de gradiente, realizados en laboratorio, se utilizan a menudo para inferir las preferencias climáticas de los animales en la ausencia de otras variables. Sin embargo, la capacidad de extrapolar los resultados de laboratorio al campo depende de la acumulación de datos ecológicos de esos organismos. Cuando la variable cuantificada se interpreta como “preferencia” térmica, existen ciertas suposiciones, a saber que, el organismo selecciona una temperatura particular por termotaxia positiva. Los anfibios, así como la mayoría de ectotermos, tienden a ser termoconfomistas, por lo que las conclusiones de experimentos con gradiente térmico tienen un significado diferente al que tienen para organismos como los ectotermos heliotérmicos, que tienen un menor rango de temperatura corporal en el laboratorio y en el campo. Evaluamos si y cómo la salamandra de espalda roja del este (*Plethodon cinereus*) se comporta cuando está presente en un gradiente térmico de arena en comparación con un control (homotérmico) arena. Las salamandras en el control de arena se desplazaron de forma clara hacia uno u otro extremo de la arena. Encontramos que las salamandras respondieron al gradiente térmico, pero su comportamiento termorregulador se limitó a evitar el extremo más caliente del gradiente, y no a una termotaxia positiva hacia una temperatura térmica que podríamos asumir como “preferida”. Nuestros resultados invitan a tomar consideraciones en como las medidas de comportamiento en laboratorio se relacionan con los comportamientos predichos de los organismos en ambientes naturales, y una reevaluación en la terminología utilizada para describir los comportamientos de movimiento en los gradientes térmicos.

## Resumo

Gradientes térmicos são comumente utilizados em estudos envolvendo organismos ectotérmicos para uma variedade de perguntas científicas. Esses experimentos realizados em laboratório são frequentemente usados para estimar a preferência térmica de animais na ausência de outras variáveis. Porém, a capacidade de extrapolar os resultados do laboratório para o campo depende do acúmulo de dados da ecologia do organismo em questão. Quando a variável quantificada é interpretada como “preferência”, há algumas premissas implícitas, incluindo que o organismo seleciona uma temperatura preferencial específica através de termotaxia positiva. Anfíbios, assim como a maioria dos ectotérmicos, tendem a ser termoconformadores. Portanto, conclusões de experimentos em gradientes térmicos carregam significados diferentes do que em organismos que mantém uma amplitude térmica estreita tanto em laboratório quanto em campo, como ectotérmicos heliotérmicos. Nós testamos se e como a salamandra *Plethodon cinereus* reage a um gradiente térmico em comparação a uma pista controle (sem variação térmica). Os resultados mostraram que as salamandras na pista de controle se moveram em direção a uma das extremidades da pista, apesar de não haver variação na temperatura. Nós observamos que as salamandras de fato exibiram respostas ao gradiente térmico, mas a termoregulação observada se limitou a evitar o lado mais quente do gradiente, e não em termotaxia positiva em direção a uma temperatura específica como seria esperado de uma temperatura dita “preferencial”. Os nossos resultados encorajam uma maior consideração de como comportamentos medidos em laboratório correspondem a previsão de comportamentos em contextos naturais, e uma reavaliação da terminologia usada para descrever comportamentos de movimentação em gradientes térmicos.

## Introduction

Temperature influences function and homeostasis in ectothermic animals, with impacts on physiological processes and spatial distributions (Angilletta Jr. 2009). However, the degree of thermal dependence varies among species, as many lineages can thermoregulate their body temperature using behavior, with great diversity of strategies and limits, according to species, ecological context, and type of thermal heterogeneity (Muñoz 2022; Dubiner et al. 2024; Giacometti et al. 2024). This assortment of thermoregulation strategies matters because the nature of temperature selection in heterogeneous thermal landscapes influences selective pressures on thermal physiology, in turn affecting evolutionary patterns (Muñoz and Bodensteiner 2019). Thermoregulatory behaviors must be properly assessed, as they are relevant for proposing, interpreting, and informing models for vulnerability under climate change (Huey et al. 2012; Kearney and Porter 2017). This scenario highlights the importance of permanently revisiting the methods used to assess behavioral thermoregulation, often consisting in analyzing the behavior of target specimens in a thermal gradient spanning a certain range of temperatures. The data, in this case, are substrate or body temperatures recorded periodically, and the central tendencies of these data are used to infer behavioral preferences and patterns.

Many studies seek to connect laboratory-estimated thermal behavior to that of their free-ranging counterparts. This inference requires discriminating between what animals can do, as tested in a laboratory, and what animals will do in the field (Fry 1947; Navas et al. 2021). Such inference is straightforward when field behavior is well known; small, heliothermic lizards, for example, tend to display strong and consistent thermotaxis in laboratory studies, and this behavioral pattern is often replicated in the field (Huey and Stevenson 1979; Hertz et al. 1993; Gunderson and Leal 2016; Muñoz and Losos 2018). However, behavior must also be studied in species for which little information is available, and no generalization applies to all ectotherms. Here, we use an experimental framework to explicitly test some common assumptions when performing thermal preference experiments in a lungless amphibian, the Eastern red-backed salamander (*Plethodon cinereus*).

If the objective of a given laboratory intervention is to learn about behavior in the field, then the correspondence between field and laboratory behavioral patterns becomes crucial. However, this association is intricate, and amphibians offer an excellent example to learn more about these complexities. For example, salamanders in the family Plethodontidae (lungless salamanders) respond nonrandomly to laboratory variation in temperature (Feder and Pough 1975; Feder 1982), pH (Wyman and Hawksley-Lescault 1987; Sugalski and Claussen 1997), and soil humidity (Sugalski and Claussen 1997), but when pH and humidity covary, salamanders may gravitate more strongly toward higher pHs (Sugalski and Claussen 1997), despite field data showing humidity as a key factor guiding activity patterns and spatial distributions (Heatwole 1962; Ficetola et al. 2018; Farallo et al. 2020). Laboratory behavior in response to temperature may vary with relative humidity (Galindo et al. 2018), and the body temperature of field animals may disagree with laboratory thermal selection, even under favorable environmental conditions (Feder 1982). Therefore, deducing field behavior from experimental data is not straightforward, as field distributions may reflect many behavioral drives, interactions, and constraints that are not replicated in most laboratory settings (Navas et al. 2021). Even a compelling corroboration of thermotaxis, using temperature as a single variable, does not necessarily grant reproducibility in field settings. In summary, inferring thermal ecology from whole-animal experiments is a complex analytical process that is enhanced by attention to experimental detail, and information on natural history (Bartholomew 1986).

Concerns about inference likely explain persistent cautionary advice in the literature regarding both methods and, perhaps more strongly, interpretations. Concerning critical thermal limits (for ectothermic vertebrates this is typically defined as the lowest and highest temperature at which locomotor capacity ceases, determined by the inability of animals to right themselves when flipped onto their backs), for example, articles directly consider the costs and benefits of different methodological approaches (Ørsted et al. 2022) and the analytical consequences of behavioral vs physiological endpoints (Ribeiro et al. 2012; Lutterschmidt and Hutchison 1997a, 1997b). Parallel caveats extend to vulnerability indices based on critical temperatures, emphasizing the need to make underlying assumptions explicit (Clusella-Trullas et al. 2021) and the consequences of the scale at which the analysis occurs, either in time or space (Garcia et al. 2019). With respect to thermal gradients, the discussion emphasizes the importance of experimental controls (Hutchison et al. 1966), the methods to report variance (Feder 1982), the implications of metaphors (such as “preferred,” “selected,” or “eccritic”) in variable naming (Pough and Gans 1982), and the need to restrict hypotheses about thermotaxis to its appropriate inference space (Navas et al. 2021).

Here, we designed and applied a thermal gradient method to evaluate temperature selection in small animals. We had the goal of both evaluating the nature of the behavior of salamanders in relation to temperature under an experimental setting, as well as testing the methodological factors that can influence inference. We focused on the Eastern red-backed salamander (*Plethodon cinereus*), a lungless, terrestrial amphibian found across Eastern North America, northward from North Carolina to southern Ontario, and westward from the Atlantic Coast to Minnesota. This species has been widely studied, is abundant and easy to handle, and performs well under laboratory conditions (Jaeger et al. 2016). Focusing on *P. cinereus*, we asked, “*Does the behavior in an experimental arena change with exposure to a thermal gradient? And, if so, how does behavior change?”* In parallel, we ask three methodological questions: *(1) What is the usefulness of a control in thermal gradient experiments? (2) To what extent do small changes in the setup of an experimental thermal gradient influence results?* and *(3) What confounding variables are likely to affect results and require caution?* The last two questions relate to variables related to the orientation and location of the experimental setup, as described in the Methods, and to changes in the temperature range*.* Question (1) relates to the need for a control, which is inherent to most biological questions to be answered experimentally. Because our question concerning salamander behavior uses the term “change,” it requires a comparative framework. We use the control arena for this purpose, where salamanders experience the same conditions as in the thermal gradient, but under uniform room temperature. We aimed for strong inference in our biological question and designed a “crucial experiment” (Platt 1964) focusing on patterns of movement. Potential results could range from no movement (no experimental signal) to random movement (signal unrelated to temperature, the main variable tested), although we expected animals to move, and temperature to have some impact on behavior. Based on this expectation, we formulated five alternative hypotheses and associated predictions involving random movement and specific nonrandom behaviors related to a temperature gradient. Our hypotheses and predictions appear in Table 1 and are related to the behavioral response variables described in “Methods.” Our research also includes inductive components, as we had no *a priori* hypotheses regarding how certain variables (e.g., Collection Date or Body Mass) could affect behavior. Notably, our aim is not to propose and describe an ideal thermal gradient experiment, but rather to explore the method with the advantages of a common species that responds well in captivity, and that might be generalizable to other thermoconforming species. Thus, we can enhance information from repeated measures, and the use of individuals as their own controls. The setup we used may be impractical, even undesirable, under several contexts, but our intention is to illustrate what we learned from each variable quantified, so that researchers find inspiration to decide what is important for their context-specific cases and conditions.

**Table 1.** Hypotheses (column heads) tested, and predictions based on variables (italics, first column)

The behavior of salamanders in a thermal gradient is:	Unrelated to temperature	Warm avoidance	Cold avoidance	Avoidance of both extremes	Indicative of keen thermoregulation
*Reaches to number 22 (warmest)*	Similar	Fewer	Similar	Fewer	Fewer
*Reaches to number 1(Coldest)*	Similar	Similar	Fewer	Fewer	Fewer
*Coast to coast events (1* *–* *22)*	Similar	Fewer	Fewer	Fewer	Fewer
*Dominant position in the gradient*	Similar	Fewer at high values (>20)	Fewer low values	Fewer extreme values	Fewer in a range to be determined, probably on the colder side.
*Total distance moved*	Similar	Somewhat lower	Somewhat lower	Lower	Lower
*Motionlessness*	Similar	Lower at high release number	Lower at small release number	Lower at high and small release numbers	Lower
*Maximum number visited*	Similar	Lower, less frequent >20	Similar	Lower, less frequent < 3	Lower
*Minimum number visited*	Similar	Similar	Fewer or less frequent	Fewer or less frequent	Similar or higher
*Time at last movement*	Similar	Similar or earlier	Similar or earlier	Possibly earlier	Earlier
*Number of movement events*	Similar	Somewhat fewer, related to release number.	Somewhat fewer, related to release number.	Fewer as a function of release number	Fewer
*Body temperature (extrapolated in control)*	Similar	Lower	Higher	Lower	Lower variance more than anything else.

Comparative statements are Gradient animals relative to Control.

## Methods

### Experimental setup

#### Apparatus

We built four experimental units, each one consisting of two rectangular cases (110 cm long and 9.5 cm wide) termed *arenas*, one of which was activated with a thermal gradient while the other remained at the room temperature of 15°C (*control*). To keep track of the position of animals within each arena, we split and labeled arenas into 22 segments (hereafter, “positions”), approximately 4.5 cm each, with numbers printed in one detachable label placed on the wall behind the units. For logistic reasons, two controls and two gradients were always paired together (Fig. S1).

#### Substrate

We originally tried layer-type substrates (paper towels and gauze), but salamanders occasionally hid under the material, which also had to be washed or replaced after each use to minimize pheromone marking, a characteristic of *Plethodon* salamanders (Jaeger and Forester 1993). We switched to a 1 cm depth layer of fine vermiculite, a substrate that could be mixed after each test, homogenizing eventual chemical cues. Individuals occasionally moved their bodies or limbs in what looked to be digging behavior, but no salamander was able to fully hide under the vermiculite.

#### Heating and cooling

Heat was generated by placing a heating cable under the first half of each gradient; the cable was more densely distributed at the extreme and decreased in density toward the middle, where it exited the gradient. To diminish lateral gradients or sharp changes associated with the exact position of the heating cable, we added a copper sheet between the gradient and the heating cable so that the heating cable was secured to the copper sheet with electrical tape. The second half of the gradient was not heated, and the final portion (positions 1–4 according to the labeling code) rested over crushed ice placed on a Styrofoam cooler (contact area space 21 × 19 cm, depth 16 cm). During experiments, we added ice as needed to compensate for melting.

#### Thermal structure of gradients

**Fig. 1.**
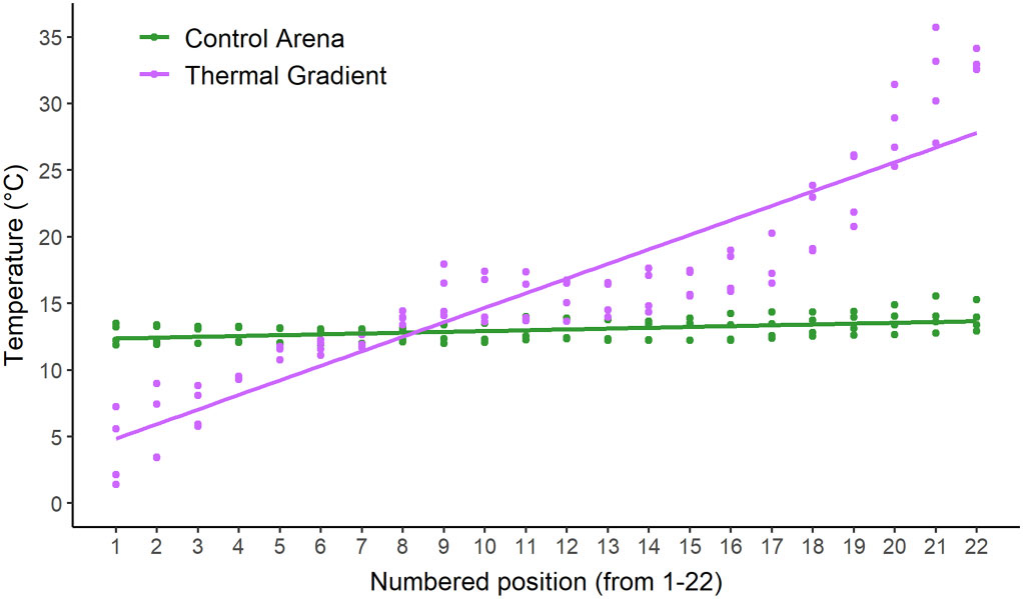
Baseline thermal structure of control and thermal gradient arenas along numbered positions. Gradient and control temperatures were comparable at positions 9–14.

When building the gradient, we aimed for balance among three factors. First, we deemed it important to retain the behavioral information generated by extreme temperatures and opted for a minimum temperature approaching 5°C (mean ± SD: 5.47 ± 2.91°C, min: 0.5°C, max: 13.4°C) and a maximum temperature of about 33°C (mean ± SD: 33.12 ± 4.30°C, min: 26.2°C, max: 45.6°C). Second, we wanted to avoid overly long or short gradients because the temperature selected by urodeles in thermal gradients may be influenced by the length of the gradient relative to the body length of specimens (Navas et al. 2021), and so opted for arenas of 110 cm in length. Lastly, and despite the impact of the air flow inside an environmental chamber, we decided to perform experiments in the regulated environment of a walk-in environmental chamber (15°C) instead of a laboratory bench at room temperature. The resulting gradient met target temperature values on the ends, although its middle part (positions 10–12) was unavoidably influenced by airflow. The thermal structure of the baseline gradient appears in Fig. 1. The gradient was constant within a given experimental day, but we allowed experiment-wise minor changes in the minimum and maximum temperature to evaluate possible impacts. Also, on each experimental day we recorded the minimum, maximum, and central temperatures of each gradient. A thermographic image of gradients and controls is presented in Fig. S2.

#### Variables related to orientation and location of the experimental setup

The walk-in environmental chamber had two side-to-side stands, each one supporting two parallel experimental units (two control arenas and two thermal gradients; Fig. 1). Because air flow, light, and air temperature varied inside the chamber, we took measures thought to reduce, or at least turn quantifiable, the impact of such unplanned sources of variation. Regarding light and air flow, we applied red translucent plastic over the light sources (located in the ceiling in the center of the room above roughly the middle of the gradients) and placed panels that reduced direct air flow on experimental units. Despite these actions, we could not eliminate the impacts of the location and orientation of experimental units and thought it desirable to alternate the position and orientation of gradients and controls. Thus, we created a systematic protocol to rotate the position and orientation of gradients and controls.

**Fig. 2.**
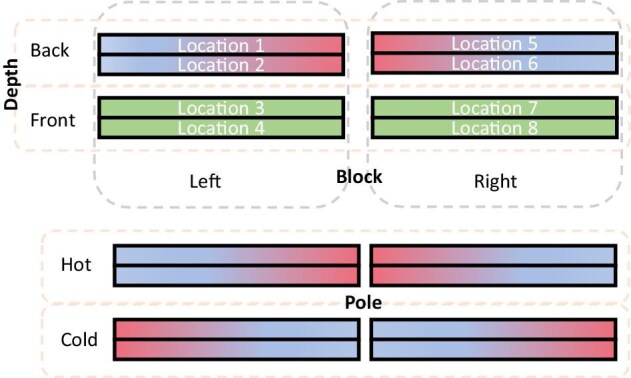
Illustrative schematic of the setup variables and their states. Block relates to position of arena: Left or Right of the room; Depth represents the position of arenas in relation to observer, Back being further from the observer and Front, closer; Pole refers to the orientation of the hot end of the gradient, which for gradients in both right and left sides faced either toward the middle of the room (Hot) or facing away from each other towards the walls (Cold).

Our protocol involved three orientation categorical variables (see diagram in Fig. 2). Each arena occupied one out of eight possible locations, and these different locations were subject to different spatial effects of the room. Therefore, we assigned Block, a variable with two states, Right (location 1–4) and Left (location 5–8), referring to the right or left stand inside the chamber. At each such stand (Block, hereafter), because arenas were oriented perpendicular to the observer, either controls or thermal gradients could be at the front (nearer to the observer, i.e., the open floor space of the environmental chamber), or back (further from the observer, i.e., nearer the wall of the chamber) and this aspect of location was termed Depth, with two states, Front and Back. For practicality, either cold or hot extremes met in between the two blocks, and we associated these two possible orientations to a third variable, Pole, with two states, Cold and Hot. Therefore, a Cold state was defined when the gradients at the left block ran hot to cold (from left to right), whereas those at the right block ran cold to hot, and consequently, both sets of gradients were closest at their colder extremes, between the two stands (Fig. 2). Conversely, a Hot state was the opposite, with both sets of gradients meeting at their hottest extreme. No other practical orientation was viable, as the hot and cold ends could not meet without serious interference with the aimed thermal ranges. We shifted from Cold to Hot state every experimental day, so that gradients would necessarily run in different directions. These variables were used exploratorily. For consistency, the hot end of gradients was always associated with arena segment 22, and control arenas followed the number scheme of neighboring thermal gradients. The specific sequence of changes applied is listed in Fig. S3.

### Animals and data gathering

#### Animal collection

This study included 24 eastern red-backed salamanders (*Plethodon cinereus*) collected between September and November 2023 in New Haven, CT, mainly at East Rock Park and neighboring areas. These salamanders were collected in three groups of eight individuals, from September (first group) to November (third group). While in the field, we placed salamanders in individual plastic bags that were filled with moist leaf litter, and salamanders remained there until the next day. Then, we transferred the specimens to individual plastic cases containing humid paper towels, which were maintained in a walk-in environmental chamber set at 15°C. We fed salamanders weekly with flightless fruit flies (*Drosophila*) and applied established husbandry protocols (e.g., Reiter et al. 2014).

#### Temporal distribution of data collection

With a few exceptions and pauses associated with the academic calendar, we ran experiments twice a week from September to December 2023. During this time, we tested each of the 24 salamanders five times. On the first experimental day for a given salamander group, we individually placed four salamanders in the control arenas (room temperature set at 15°C) and four in the thermal gradients. On the next day of experiments, the salamanders that were previously assigned to the control were assigned to the thermal gradient, and vice versa. We repeated this procedure over two additional experimental days. Then, on one additional experimental day, we tested four salamanders as control and four as experimental. In total, all salamanders were tested twice as control, twice as experimental, and once as either control or experimental. We did not attempt to test all salamanders six times, to guarantee that all 24 individuals would be studied during the natural surface activity season (Anthony and Pfingsten 2013; Fisher-Reid et al. 2024). Experiments were concluded by mid-December 2023.

### Experimental protocol

To start an experiment, we randomly defined the position (1–22) in the gradient at which each salamander would be released and placed a wet cotton circle at the designated spot. Next, we carefully placed salamanders under the cotton circles, aiming to reduce movement until the onset of formal observations. Once all salamanders were in place, we defined time zero and activated a stopwatch. We collected behavioral data throughout the experiment (*e.g*., number of end-to-end movements and visits to the extreme segments of arenas; see below) and every 5 min (standard variables, e.g., numbered position in arena and salamander dorsal temperature; see below). The experiment continued for 105 min and all tests started between 12:00 and 14:00 h (EST). On two occasions (27 September and 04 October), experiments were finished at 95 and 80 min, respectively, to accommodate experimenters’ schedules; however, the amount of movement was typically small later in experiments. There were three-four people present in the walk-in environmental chamber during data collection: this team comprised two observers, each watching four of the thermal gradients, one note taker responsible for writing the information, and one person who assisted the observers. *Plethodon cinereus* is primarily nocturnal, but individuals are also surface-active during the daytime during favorable weather (Anthony and Pfingsten 2013) and were thus expected to respond to thermal cues during our experiments. Every 10 min, all arenas were sprayed with mineral water to keep the substrate humid and the animals fully hydrated. Although spraying water may momentarily change the temperature of salamanders and gradient, we thought that preferable to dehydration, since hydric stress has been shown to affect salamander selected temperature (Galindo et al. 2018).

### Variables quantified

Our primary goal concerned how salamander position and movement differed between the control arenas and thermal gradients, and which of the hypotheses concerning salamander behavior summarized in Table 1 were supported. In addition, we recorded several variables related to salamanders and experimental setup to explore their effect on inference.

#### Behavioral response variables

These are aspects of behavior quantified in terms of three dominant dimensions: movement, position, and temperature. The variables recorded continuously were (1) Reaches to 22 (number of independent visits to position 22, which was the warmest in the case of thermal gradients), (2) Reaches to 1 (reaches to position 1, the coldest in thermal gradients), and (3) End-to-end events (events of movement from position 1 to 22, or vice versa). Variables registered every 5 min were (4) Position in gradient, (5) Orientation (the dominant direction of the head of salamanders according to a cardinal plane with four values only), and (6) Salamander dorsal surface temperature (hereafter, body temperature; T_b_) measured with an infrared thermometer (General IRT207) aimed at the dorsum on the mid-body of the salamander at a distance of approximately 20 cm from the salamander (distance-to-spot ratio of 8:1 inches). Body temperature represents a function of room oscillation in controls, and of salamander position in gradients. From these measurements we also determined (7) Movement (the number of 4.5 cm segments traversed; e.g., a salamander at position 8 found 5 min later at position 6 would have a count of “2”), (8) Motionlessness (number of consecutive observations at the same position), (9) Maximum position visited (equivalent to hottest reach in thermal gradients), (10) Minimum position visited (equivalent to coldest reach in activated gradients), (11) Time at last movement (experimental time at which salamanders moved for the last time), and (12) Number of movement events (count of events in which the position was different from the previous observation).

#### Experimental setup variables

We also measured variables that, according to published observations, may influence results. These are (1) Minimum Gradient Temperature, (2) Maximum Gradient Temperature, (3) Gradient Temperature Range, and (4) Salamander release position (according to labels). In addition, we measured setup variables that were not a part of our conceptual framework but could influence results and require care. These include the already mentioned variables: (5) Block, (6) Depth, (7) Pole, plus (8) Mean control temperature, and (9) Mean control variance. The last two mentioned are useful because they reflect unavoidable oscillations of room temperature that changed slightly across experimental days.

#### Salamander variables

Variables related to the collected salamanders that could influence results. These are (10) Capture date, (11) Days to test (between capture and test day), (12) Order of testing, (13) Sex, and (14) Body mass (g).

### Statistical analyses

To test which of our proposed hypotheses were supported by the data, we ran a series of models, one for each of the response variables listed in Table 1, comparing control arenas and thermal gradients. The statistical model chosen for each variable was selected based on variable type (numerical, ordinal, count) and the evaluation of whether variables conformed to assumptions of the models. When applicable, we used salamander identity as a random effect. Specific models used and their hypothesized patterns are listed in Table 2. We report variance explained for the fixed effects and the full model using the marginal and conditional R^2^, respectively, obtained from function *r.squaredGLMM* implemented in the R package “MuMIn” (Nakagawa and Schielzeth 2013).

**Table 2.** Pattern of behavioral response variables of salamanders in thermal gradient in relation to the controls

Behavioral response variables	Pattern	Mean control (SD)	Mean gradient (SD)	*P*-value	Model	R²m	R²c
*Reaches to number 22 (warmest)*	Lower	1.03 (1.18)	0.70 (1.11)	0.07	GLMM	0.024	0.12
*Reaches to number 1(Coldest)*	Higher	1.17 (1.39)	1.65 (1.49)	**0.036**	GLMM	0.028	0.277
*Coast to coast events (1* *–* *22)*	Lower	0.93 (1.56)	0.90 (1.56)	0.28	GLMM	0.012	0.479
*Mean position in the gradient*	Lower	9.64 (7.80)	5.38 (5.22)	**<0.001**	GLMM	0.095	0.123
*Total movement*	Lower	32.02 (37.79)	28.62 (25.08)	0.763	LMM	0.095	0.123
*Motionlessness*	Lower	16.33 (3.87)	16.18 (3.48)	0.77	LMM	0.001	0.089
*Maximum number visited*	Lower	17.45 (6.37)	16.12 (5.53)	**0.021**	Ordinal	NA	NA
*Minimum number visited*	Lower	3.57 (5.22)	2.65 (3.90)	0.28	Ordinal	NA	NA
*Time at last movement*	Lower	44.83 (34.76)	41.17 (33.41)	0.56	LMM	0.003	0.003
*Number of movement events*	Higher	4.20 (3.30)	4.35 (3.20)	0.71	GLMM	0.001	0.104
*Body temperature (extrapolated in control)*	Lower	15.20 (10.44)	9.91 (5.64)	**<0.001**	LMM	0.09	0.135

Pattern refers to whether the behavioral response value was higher or lower in the thermal gradient relative to the control. When applicable we report the marginal (R²m) and conditional (R²c) R^2^ for the LMMs and GLMMs.

GLMM = generalized linear mixed-effects model; LMM = linear mixed-effects model; Ordinal = ordinal regression.

#### Movement

To investigate salamander movement in our experimental system, we took three different strategies: First, we evaluated if treatment (control or thermal gradient) affected total movement of the salamander throughout the trial. We measured movement as “total distance moved” by counting the number of segments of the arena traversed by the salamander every 5 min and summing them all for the entirety of the trial (see section “Behavioral response variables” for details). We used linear mixed effects model (LMM) with treatment (control arena vs. thermal gradient), release position (1–22), salamander mass, and sex as fixed effects, and salamander identity as random effect to account for repeated measurements. We used the R package “nlme” (Pinheiro and Bates 2000) for this and similar analyses. Salamander’s total movement was log-transformed to conform to the assumption of normality of residuals. Because of one trial in which the salamander had a movement of zero, we added 1 to all values before log-transforming. Running the analysis without log-transforming or removing the 0 value resulted in qualitatively identical results. Sex and mass were not found to significantly affect movement or other response variables, so were excluded throughout.

Secondly, we used a Komolgorov–Smirnov test to compare whether movement distributions along time periods were equivalent between control and gradients. Lastly, to test whether there were individual differences in movement between salamanders, we used a repeatability estimation with a Poisson distribution using the function *rpt* implemented in the R package “rptR” (Stoffel et al. 2017). This analysis tests if the amount of variation within individuals is smaller than variation among individuals, which is evidence for behavioral repeatability. We quantified repeatability using the intraclass correlation coefficient (*R*) and considered behavior repeatable if the 95% confidence intervals did not overlap zero.

#### Salamander position

To determine if salamander position in thermal gradients differed from controls, we used LMM with mean position as the response variable, treatment as a fixed effect, and salamander ID as a random effect to account for individual differences. We initially also included salamander mass, sex, and release position as covariates, but since they did not have significant effects, we removed them from the final model.

Within each of the treatments, we also ran a chi-square analysis to determine if salamanders were randomly distributed across numbered positions in the arenas, or if their observed positions were different than expected by chance. For those analyses, we counted the number of salamanders for which the final position corresponded to each numbered position (1–22) in control and thermal gradients separately and then ran a chi-square analysis on each treatment to determine whether the salamander position was significantly different from a random (uniform) distribution across positions.

#### Salamander body temperature (T_b_)

We ran a LMM with average temperature for the whole duration of the trial as the response variable and treatment (control or thermal gradient) as a fixed effect with salamander ID as a random effect. We implemented a model in the R package “nlme” that accounts for heteroscedasticity since the thermal variance between treatments was different. Running the model without accounting for heteroscedasticity yields qualitatively equivalent results. We ran an additional LMM estimating what control temperatures would be if salamanders were in thermal gradients. We did that by obtaining an average temperature for each of the numbered positions in the gradients (as shown in Fig. 1) and then assigning these temperatures to control salamanders based on the positions they were at in each time period. This allowed us to compare actual T_b_ in thermal gradients with T_b_ salamanders would have had in control arenas if they were thermally heterogeneous.

#### Influence of setup variables on salamander T_b_ and position

The full list of variables considered can be found in the “Variables quantified” section of the methods. We used linear mixed effects models (LMMs), with salamander ID as a random effect to account for repeated measures, and either T_b_ or salamander average position (separately) as response variables. We ran separate models depending on the predictor variables as described below.

To determine the effect of exploratory variables relating to the gradient structure, (release position, and minimum, maximum, and breadth of temperature in the gradient) we considered only data from thermal gradients. Because thermal breadth was highly correlated with minimum (Pearson's r = −0.66, *P* < 0.001) and maximum temperature (Pearson's r = 0.86, *P* < 0.001), we ran one model that included gradient thermal breadth and release position as predictors and another with minimum and maximum temperature, and release position as predictors. We log-transformed T_b_ to conform to model assumptions of the normality of residuals.

To test for the effect of additional setup variables on average T_b_, we ran linear mixed effects models with salamander ID as a random effect. We first ran one global model including Treatment (control or thermal gradient), Block (Left or Right), and Depth (Front or Back) as fixed effects. We removed Pole (Hot and Cold) from this analysis because this variable isn't meaningful in controls (since it refers to the direction of the cold and hot ends), therefore would yield a spurious interaction with treatment by default. We tested for interactions between predictors and subsequently dropped each interaction effect that was not significant, removing interactions with *P*-values > 0.05 sequentially, until only significant interactions were left. We repeated this process with each treatment separately, including Pole as a variable in the analysis of thermal gradients. We performed post-hoc tests with the estimated marginal means implemented in the R library “emmeans” (Lenth 2017).

## Results

### Salamander movement and position

**Fig. 3.**
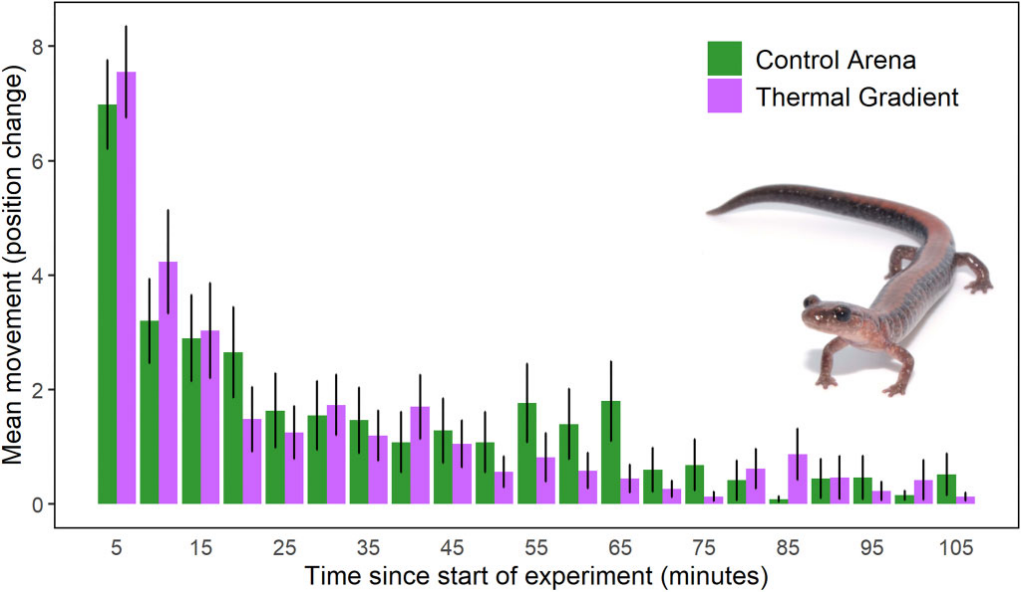
Bar graph showing salamander mean movement through time (minutes since beginning of trial), error bars correspond to standard errors. Movement was defined as the change in position according to numbered sections of arenas. Each numbered section has a width of approximately 5 cm, so each movement unit corresponds roughly to 5 cm.

Although some individuals remained motionless most of the experimental time, both control and treatment groups had nonzero movement, particularly at the beginning of the experiment (Fig. 3). Therefore, the hypothesis “No movement” requires no further consideration. Salamanders in both gradients or controls tended to move more often over the first 20 min of experimental time (Fig. 3) and displayed a similar distribution of movement over time (Kolmogorov–Smirnov test D = 0.24, *P* = 0.59). Salamanders in thermal gradients moved a median of 21.5 segments (mean = 28.62; max = 96; min = 1), and the counterparts in controls moved a median of 20.5 units (mean = 32.02; max = 185; min = 0). Control and gradient salamanders were comparable in the log-transformed total movement throughout the trials (estimate = 0.05, *t*-value_1,94_ = 0.30, *P* = 0.76; Table 2), and we found a weak but significant effect of initial position on total movement (estimate = 0.03, *t*-value_1,94_ = 2.18, *P *< 0.05), with salamanders moving slightly more when initially placed on higher positions (warmer side on thermal gradients). Ranges for all variables referenced in Table 1 are reported in Table S1.

**Fig. 4.**
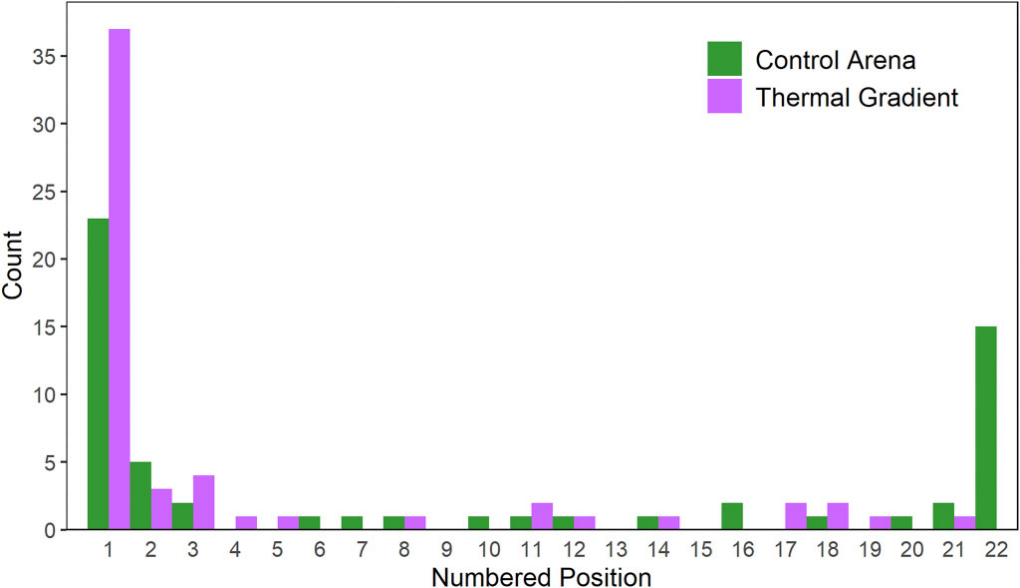
Histogram of salamander median position in arenas (as represented by numbered sections); in thermal gradients lower numbers are always associated with the colder temperatures. Note that in control arenas salamanders tended to remain in both ends of the gradient (positions 1 and 22), while salamanders in thermal gradients remained overwhelmingly more on position 1, which corresponds to the cold end.

**Fig. 5.**
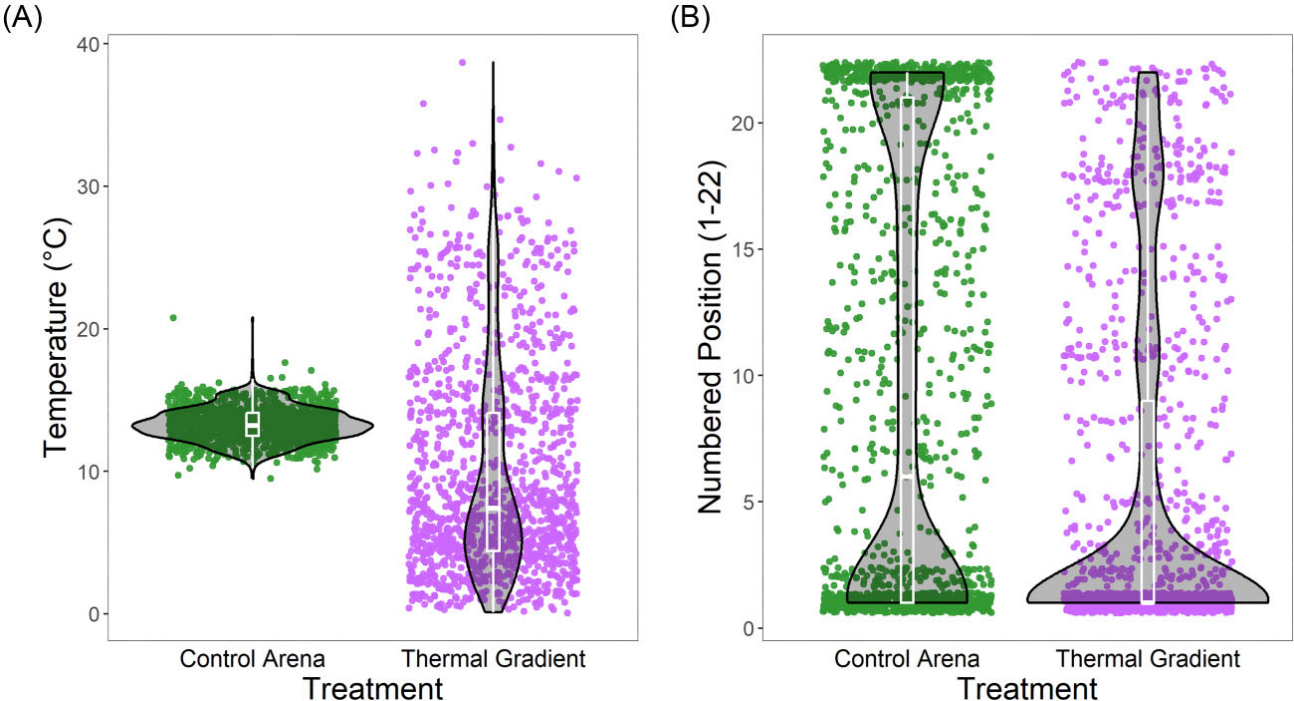
Violin plots showing temperature (**A**), and numbered position (**B**) of salamanders in control arenas and thermal gradients. Each point represents one measurement, so each salamander is represented by several points, for as many times it was measured during one trial. Note that salamanders in the thermal gradient exhibited lower temperatures on average than controls (**A**), this is likely explained by the tendency of salamanders to remain at the ends of the arenas, in both extremes (positions 1 and 22) in control arenas, and only on the cold end (number 1) in thermal gradients.

Despite the equivalent amount of movement between treatments, the thermal gradient clearly influenced the behavior of salamanders in the system, particularly from the perspective of the specific positions occupied. In both treatments, salamanders were distributed nonrandomly (control arenas: chi-squared = 321.33, df = 21, *P* < 0.001, thermal gradient: chi-squared = 462.13, df = 21, *P* < 0.001) and spent more time at the extremes of the arenas (e.g., around positions 1 and 22). However, the distribution of animals in thermal gradients was unimodal and skewed toward 1 (colder end), whereas that of animals in the control arenas was bimodal, toward both 1 and 22 (Fig. 4), which is expressed by salamanders in thermal gradients occupying a lower average position (estimate: −4.24, *t*-value_1,95_ = −3.55, *P* < 0.001). For the control, records at position 1 or 22 constituted 62.31% of total observations (38.47% at 1 and 23.84% at 22). By contrast, 56.81% of the records for salamanders in thermal gradients were at position 1 (cooler end), and only 2.40% at position 21–22 (warmer end) (Fig. 5B).

### Salamander body temperature (T_b_)

As expected, given the available thermal landscape, control and experimental salamanders differed in average T_b_ (estimate: −3.40, *t*_1,119_ = −4.60, *P* < 0.001), with control animals reflecting room temperature and its variation in time and space (mean ± SD = 13.31°C ± 0.99, range = 9.5°C–20.8°C) and experimental counterparts ranging from 0.1 to 38.7°C (virtually the full range of temperatures available across all tests), with a lower average temperature (9.91°C ± 5.64) (Fig. 5A). In the thermal gradients, the average maximum T_b_ experienced by salamanders throughout a trial was 22.59°C ± 7.23, and the average minimum T_b_ was 6.04°C ± 5.34.

For further analysis, we compared the actual T_b_ of salamanders in the gradient with the temperatures that controls would have had in a thermal gradient (using the average temperature in each position from gradients as shown in Fig. 1). In thermal gradients, salamanders displayed a median T_b_ of 8.82°C and a mean of 9.91°C ± 5.64°C, whereas control salamanders would have had substantially higher counterpart values of 11.53°C and 15.20°C ± 10.44°C (based on average gradient values) (estimate = −5.27, *t*-value_1,95_ = −3.51, *P* < 0.001). This analysis corroborates the behavioral differences among groups, and the influence of activating a thermal gradient on salamander behavior, providing evidence that salamanders in thermal gradients are thermoregulating.

#### Experimental setup variables

The impact of setup variables is summarized in Tables 3 and 4. The directionality of gradients affected salamander behavior so that the variable Pole had significant effects on both salamander T_b_ and position (Table 3). Salamander T_b_ in thermal gradients was higher under a Cold Pole state (11.96°C ± 6.54) compared to a Hot equivalent (8.35°C ± 4.32). Simply, salamanders chose warmer temperatures when the hot end of thermal gradients was facing away from the middle position of the environmental chamber. Salamander position tended to be higher on the Cold state of Pole (9.27 ± 7.28) in comparison to the Hot state (5.96 ± 6.30). Since high-numbered positions always represented higher temperatures on gradients, that means that salamanders tended to move away from the middle of the room.

**Table 3.** Effects of setup variables on mean salamander body temperature in thermal gradients, and on mean salamander position in control arenas and thermal gradients

Body temperature	Fixed effects				
*System*	Value	Std. error	DF	*t*-value	*P*-value
Block	−1.44	1.38	31	−1.04	0.3
Depth	−1.53	1.35	31	−1.13	0.27
Pole	−**3.79**	**1.5**	**31**	−**2.52**	**<0.05**
Average control temperature	−0.93	1.24	31	−0.75	0.46
Control temperature range	−0.83	2.38	31	−0.35	0.73
Position	Fixed effects				
*System*	Value	Std. error	DF	*t*-value	*P*-value
Type	−**4.1**	**1.19**	**90**	−**3.44**	**<0.01**
Block	−1.1	1.2	90	−0.92	0.36
Depth	−0.72	1.24	90	−0.58	0.56
Pole	−**3.1**	**1.32**	**90**	−**2.35**	**<0.05**
Average control temperature	0.22	1.04	90	0.21	0.84
Control temperature range	0.48	2.14	90	0.22	0.82

**Table 4.** Effects of salamander variables on mean body temperature in thermal gradient salamander (top) and mean position in control and gradient salamanders (top)

Body temperature	Chisq	DF	*P*-value
Salamander group	5.59	2	0.06
Order of testing	4.44	4	0.35
Sex	3.68	2	0.16
Body mass	0.03	1	0.87
Position	Chisq	DF	*P*-value
Salamander group	1.67	2	0.43
Order of testing	4.04	4	0.4
Sex	2.68	2	0.26
Body mass	1.22	1	0.27

Since salamander Days to Test and Order of Testing were highly correlated (Pearson's r = 0.93, *P* < 0.001), we dropped the variable Days to Test from the model and kept only Order of Testing. Because the salamander Capture Date consisted of three discrete capture events, we converted that variable to a categorical variable called the Salamander Group, with three levels. Results of salamander variables are summarized in Table 4, with no significant effects on either T_b_ or position. Salamander Group had a marginally significant effect on salamander T_b_ (*P *= 0.06), possibly because the minimum gradient temperature tended to be slightly colder through the season (Fig. S4).

### Individual variation and familiarization with the system

Because salamanders in this experiment were each tested five times, we could explore the behavioral repeatability and whether being previously presented with the thermal preference arenas affected salamander behavior. Regarding movement patterns, we observed slight, but significantly repeatable behavior among individual salamanders (*R* = 0.197, SE = 0.091, CI = [0.004, 0.362], *P* < 0.001), so that some individuals were more active and others more sedentary. This variation was consistent, irrespective of whether salamanders had been presented with a thermal gradient or a control arena. The order of trials (or previous exposure to arenas), putatively related to familiarization with the system, did not influence the amount of movement observed in salamanders (Table 4).

## Discussion

We tested alternative hypotheses about the thermal behavior of the Eastern red-backed salamander, *Plethodon cinereus*, when exposed to homeothermic (control) and heterothermic (experimental) arenas, with the goal of clarifying their behavioral responses to temperature in the laboratory. This investigation addressed three topics, specifically (1) the benefits of a control arena in thermal preference experiments, (2) the impact of changes in the experimental setup on thermal behavior, and (3) the role of confounding variables on such behavior. From our results, we can discuss the behavior of salamanders in response to a thermal gradient and offer some methodological recommendations for researchers interested in related topics. We hope that this study helps researchers interested in behavioral thermoregulation, behavioral fever, mechanistic niche modeling, and other approaches, and that it provides useful insight related to how small ectothermic animals navigate heterothermal landscapes.

### Salamander behavior in control and thermal gradients

We envisioned control tests as null models for salamander behavior in arenas with conditions that approached homothermality. This “control” behavior proved to be neither random (salamanders were not even distributed across the 22 positions) nor equivalent to the experimental counterpart (use of positions varied among treatments). Salamanders in the control arenas consistently moved from the randomly selected starting point toward any of the arena's ends, followed by a tendency to remain in those extremes. Whereas this pattern was common to both control and thermal gradient arenas, individuals in the latter group that reached the hottest temperatures in the gradient rarely remained there. In controls, salamanders were not at greater risk of thermal stress at one end than the other and so remained at either end. We see two (nonmutually exclusive) explanations for this tendency to remain at the ends of the arena in the control group: salamanders could either be attracted to the end of the gradient, be less inclined to move once ends are reached, or some combination of both. In the absence of refuges (salamanders could not excavate the vermiculite substrate as also reported in Heatwole 1960), individuals could interpret the corner at the end of the arena as a shelter, and so be less inclined to move once this location is reached. Some organisms will move toward and stay near “walls” because they are attracted by touch, a phenomenon known as thigmotaxis and demonstrated for other organisms such as cockroaches (Camhi and Johnson 1999) and rats (Barnett 1963; Treit and Fundytus 1988). This could also influence salamander behavior in our trials, although there is evidence that *P. cinereus* is not driven by thigmotaxis and is more influenced by other factors such as avoidance of light (Test 1946). Avoidance of light might have contributed to the results we observed, because salamanders were more likely to move away from the middle of the room (where the light bulb was located). Irrespective of the motivation, we are positive that a control enhanced the quality of our inference when interpreting salamander thermal behavior, and suggest that controls always be applied in studies with amphibians and other ectotherms, except when thermoregulatory behavior is very well established (Angilletta Jr. 2009; Huey et al. 2012; Muñoz and Bodensteiner 2019).

Had we limited our study to consider only behavior in thermal gradients, we might have reasonably concluded that salamanders strongly favor the coldest temperatures. However, this would have been misleading, in the sense that we know, thanks to the control, that other behavioral drives add complexity to the observed responses. Collectively, our results suggest that salamander responses are composite and include (1) exploratory behavior (more pronounced at the beginning of trials), (2) avoidance of the warmest temperatures in thermal gradients, (3) a tendency to remain at the end of arenas, and (4) inclination to remain at cold end of thermal gradients. Furthermore, the positive correlation between salamander T_b_ and minimum gradient temperature suggests that salamanders in thermal gradients remained in the coldest position available at the end, and did not choose a temperature (the temperature at position 1 was 0.5–13.4°C). Even though we performed experiments during the afternoon and *P. cinereus* is a mostly nocturnal species, we do not think that invalidates our results. Salamanders explored the full range of the gradients, moving particularly often in the first 20 min of the experiment, but often throughout the trial. In addition, *P. cinereus* can be surface-active during the day when environmental conditions are ideal (e.g., after rainfall) (Anthony and Pfingsten 2013), and therefore would still be expected to respond to environmental cues during the daytime.

Two additional issues to discuss are the concept of “preference” and the potential for these laboratory findings to predict salamander field behavior. Our results suggest preferences for a position in the system, but not necessarily for a target temperature, except the avoidance of excessively hot temperatures (Paranjpe et al. 2012; Navas et al. 2021). Heat avoidance seems consistent with field behavior because, when temperatures are too high on the surface, *P. cinereus* tend to move underground (Heatwole 1962; Farallo et al. 2020; Waldron et al. 2024). For ectothermic animals—and particularly for amphibians with permeable skin—avoiding extreme heat confers advantages by mitigating eventual stress and minimizing dehydration (Riddell et al. 2024; but see Navas et al. 2021). In these taxa, thermal behavior involves not only evasion of detrimental physiological conditions but also the maintenance of water balance (Feder 1982; Feder and Londos 1984; Galindo et al. 2018).

Finally, we observed individual variation in salamander behavior. All individuals tested display heat avoidance, but patterns differ, and were repeatable, particularly regarding the configuration of movement. Some individuals had a higher tendency to move and explore the gradient than others, and this behavior was consistent in both gradients and controls. Further studies shall define if these differences are compatible with the idea of animal “personality” (Bell et al. 2009), but at this point our data are consistent with other studies showing repeatability of behaviors such as foraging or risk-taking in *P. cinereus* (Cosentino and Droney 2016; Waldron et al. 2022; Garner et al. 2024). More work is needed to identify how these differences in individual behavior as we observe in the lab translate into fitness and intraspecific variation.

### Methodological considerations

Logistical and practical constraints will often prevent researchers from applying the full range of controls, repeated measures, induced experimental variation, and location variables that we employed. Therefore, our aim here is not to propose an ideal experimental design for thermal gradients, but rather to discuss the implications of various experimental decisions. This discussion is intended to help researchers prioritize factors relevant to their specific scientific questions, study systems, and experimental contexts. Along these lines, we first examine the influence of setup variables, as there is no ambiguity regarding the impact of the system's position within the walk-in chamber. This observation suggests the potential influence of hidden variables affecting behavior.

Our design allowed us to detect the impact of the arena's orientation (Pole) on salamander position and body temperature (Tb), but the measured variables are unlikely to be direct causes of behavioral changes. Plethodontid salamanders are known to detect gradients of humidity (Heatwole 1962; Galindo et al. 2018), light (Heatwole 1960), and soil pH (Vernberg 1955; Wyman 1988), among others. Additionally, they likely detect and react to convection (air movement), which may influence rates of dehydration (Feder 1983). Many uncontrolled or unknown factors may influence salamander behavior, introducing behavioral noise relative to the target variable, which in this case was substrate temperature. Randomizing the position of thermal gradient arenas relative to physical space is a broadly valuable recommendation, as previously suggested by other authors. Because the importance of these variables is context-dependent, we report the numerical impact of such variables, recognizing that magnitudes may be critical for some research questions but acceptable or negligible for others.

Despite thermal preferences being commonly reported for ectotherms, our results fail to corroborate this trend in *P. cinereus* and instead suggest avoidance behavior in relation to heat and confounding factors related to the physical structure of the arenas. These results have two main implications. First, they contribute to the understanding of this species, suggesting that its thermal behavior is complex and influenced by broader environmental cues, as indicated by other studies on plethodontid salamanders (Feder and Pough 1975; Feder 1982; Galindo et al. 2018). Second, our findings highlight the critical importance of controls in testing hypotheses regarding thermotaxis (e.g., active temperature selection). Central tendency values derived from thermal gradients are insufficient for a comprehensive analysis of thermal behavior (see review and additional references in Navas et al. 2021). However, we can leverage the inference we can obtain from a common salamander that is easy to work with in a laboratory context to inform studies with rare species or in limited field conditions, in which complex and time-consuming experimentation is prohibitive. Amphibians face significant risks from climate change (Wu et al. 2024), and it is therefore important that our inferences from thermal preference experiments are as informative and biologically relevant as possible.

Finally, we caution researchers about the reported effects of arena ends, particularly at the cold end of the gradient, given the observed heat-avoidance behavior. One possibility is to provide cover objects throughout the arena (Feder and Pough 1975), or to adopt circular or elliptical thermal gradients that eliminate terminal edges while retaining lateral ones (Touska et al. 2016). While this approach may be helpful if the observed response has a thigmothermic basis, circular designs do not inherently eliminate shading or other illumination-related factors and are challenging to refine. Another possibility is to use arena ends with temperatures beyond the species' tolerance range, assuming they would be avoided; however, such avoidance is not guaranteed, and animals may risk fatal overexposure to heat (Navas et al. 2007). A list of potential recommendations could become exhaustive; thus, our core advice is to conduct pilot experiments in which behavior is carefully observed, both in the presence and absence of induced thermal gradients. Within the limits of feasibility, the positions of arenas should be randomized with respect to light and airflow sources, and controls are invaluable for robust experimental design.

## Supplementary Material

obaf015_Supplemental_File

## Data Availability

The data used in the present paper are available upon request from the corresponding author (J.L.B.).

## References

[bib1] Angilletta MJ Jr . 2009. Thermal Adaptation: a Theoretical and Empirical Synthesis. New York, USA: Oxford University Press.

[bib2] Anthony CD, Pfingsten RA. 2013. Eastern Red-backed salamander, *Plethodon cinereus*. Amphibians Ohio 17:335–60.

[bib3] Barnett SA . 1963. The Rat: a Study in Behavior. New York: Routledge.

[bib4] Bartholomew GA . 1986. The role of natural history in contemporary biology. Bioscience 36:324–9.

[bib5] Bell AM, Hankison SJ, Laskowski KL. 2009. The repeatability of behaviour: a meta-analysis. Anim Behav 77:771–83. 24707058 10.1016/j.anbehav.2008.12.022 PMC3972767

[bib6] Camhi JM, Johnson EN. 1999. High-frequency steering maneuvers mediated by tactile cues: antennal wall-following in the cockroach. J Exp Biol 202:631–43. 9929464 10.1242/jeb.202.5.631

[bib7] Clusella-Trullas S, Garcia RA, Terblanche JS, Hoffmann AA. 2021. How useful are thermal vulnerability indices? Trends Ecol Evol 36:1000–10. 34384645 10.1016/j.tree.2021.07.001

[bib8] Cosentino BJ, Droney DC. 2016. Movement behaviour of woodland salamanders is repeatable and varies with forest age in a fragmented landscape. Anim Behav 121:137–46.

[bib9] Dubiner S, Aguilar R, Anderson RO, Arenas Moreno DM, Avila LJ, Boada-Viteri E, Castillo M, Chapple DG, Chukwuka CO, Cree A et al. 2024. A global analysis of field body temperatures of active squamates in relation to climate and behaviour. Global Ecol Biogeogr 33:1–18.

[bib10] Farallo VR, Muñoz MM, Uyeda JC, Miles DB. 2020. Scaling between macro- to microscale climatic data reveals strong phylogenetic inertia in niche evolution in plethodontid salamanders. Evolution 74:979–91. 32190909 10.1111/evo.13959

[bib11] Feder ME . 1982. Thermal ecology of neotropical lungless salamanders (Amphibia: Plethodontidae): environmental temperatures and behavioral responses. Ecology 63:1665–74.

[bib12] Feder ME . 1983. Integrating the ecology and physiology of plethodontid salamanders. Herpetologica 39:291–310.

[bib13] Feder ME, Londos PL. 1984. Hydric constraints upon foraging in a terrestrial salamander, *Desmognathus ochrophaeus* (Amphibia: Plethodontidae). Oecologia 64:413–8. 28311459 10.1007/BF00379141

[bib14] Feder ME, Pough FH. 1975. Temperature selection by the red-backed salamander, *Plethodon* *c. cinereus* (Green) (Caudata: Plethodontidae). Comp Biochem Physiol A Physiol 50:91–8. 10.1016/s0010-406x(75)80207-6 234066

[bib15] Ficetola GF, Lunghi E, Canedoli C, Padoa-Schioppa E, Pennati R, Manenti R. 2018. Differences between microhabitat and broad-scale patterns of niche evolution in terrestrial salamanders. Sci Rep 8:1–12. 30002477 10.1038/s41598-018-28796-x PMC6043550

[bib16] Fisher-Reid MC, Grayson KL, Grouleff SR, Hair MA, Matlaga TJH, Ireland AK, Mead LS, St John A, Starr M, Sterrett SC et al. 2024. Eastern red-backed salamanders: a comprehensive review of an undervalued model in evolution. Herpetol Monogr 38:74–121.

[bib17] Fry FEJ . 1947. Effects of the environment on animal activity. Publ Out Fish Res Lab 55:1–62.

[bib18] Galindo CA, Cruz EX, Bernal MH. 2018. Evaluation of the combined temperature and relative humidity preferences of the colombian terrestrial salamander *Bolitoglossa ramosi* (Amphibia: Plethodontidae). Can J Zool 96:1230–5.

[bib19] Garcia RA, Allen JL, Clusella-Trullas S. 2019. Rethinking the scale and formulation of indices assessing organism vulnerability to warmer habitats. Ecography 42:1024–36.

[bib20] Garner KL, Ryan JM, Tingle JL, Hickerson CAM, Anthony CD. 2024. Covariation and repeatability of aggressive and risk-taking behaviours in a terrestrial salamander (*Plethodon cinereus*). Anim Behav 213:1–10.

[bib21] Giacometti D, Palaoro AV, Leal LC, de Barros FC. 2024. How seasonality influences the thermal biology of lizards with different thermoregulatory strategies: a meta-analysis. Biol Rev 99:409–29. 37872698 10.1111/brv.13028

[bib22] Gunderson AR, Leal M. 2016. A conceptual framework for understanding thermal constraints on ectotherm activity with implications for predicting responses to global change. Ecol Lett 19:111–20. 26647860 10.1111/ele.12552

[bib23] Heatwole H . 1960. Burrowing ability and behavioral responses to desiccation of the Salamander, *Plethodon cinereus*. Ecology 41:661–8.

[bib24] Heatwole H . 1962. Environmental factors influencing local distribution and activity of the Salamander, *Plethodon cinereus*. Ecology 43:460–72.

[bib25] Hertz PE, Huey RB, Stevenson RD. 1993. Evaluating temperature regulation by field-active ectotherms: the fallacy of the inappropriate question. Am Nat 142:796–818. 19425957 10.1086/285573

[bib26] Huey RB, Kearney MR, Krockenberger A, Holtum JAM, Jess M, Williams SE. 2012. Predicting organismal vulnerability to climate warming: roles of behaviour, physiology and adaptation. Philos Trans R Soc B Biol Sci 367:1665–79. 10.1098/rstb.2012.0005 PMC3350654 22566674

[bib27] Huey RB, Stevenson RD. 1979. Integrating thermal physiology and ecology of ectotherms: a discussion of approaches. Am Zool 19:357–66.

[bib28] Hutchison VH, Dowling HG, Vinegar A. 1966. Thermoregulation in a brooding female Indian python, *Python molurus bivittatus*. Science 151:694–6. 5908075 10.1126/science.151.3711.694

[bib29] Jaeger RG, Forester DC. 1993. Social behavior of plethodontid salamanders. Herpetologica 49:163–75.

[bib30] Jaeger RG, Gollmann B, Anthony CD, Gabor CR, Kohn NR. 2016. Behavioral Ecology of the Eastern Red-backed Salamander: 50 Years of Research. New York, USA: Oxford University Press.

[bib31] Kearney MR, Porter WP. 2017. NicheMapR—an R package for biophysical modelling: the microclimate model. Ecography 40:664–74.

[bib32] Lenth RV . 2017. emmeans: estimated Marginal means, aka Least-Squares means. CRAN Contrib Packages. https://cran.r-project.org/web/packages/emmeans/emmeans.pdf

[bib33] Lutterschmidt WI, Hutchison VH. 1997a. The critical thermal maximum: data to support the onset of spasms as the definitive end point. Can J Zool 75:1553–60.

[bib34] Lutterschmidt WI, Hutchison VH. 1997b. The critical thermal maximum: history and critique. Can J Zool 75:1561–74.

[bib35] Muñoz MM . 2022. The Bogert effect, a factor in evolution. Evolution 76:49–66. 34676550 10.1111/evo.14388

[bib36] Muñoz MM, Bodensteiner BL. 2019. Janzen's hypothesis meets the Bogert effect: connecting climate variation, thermoregulatory behavior, and rates of physiological evolution. Integr Org Biol 1:1–12. 10.1093/iob/oby002 PMC7671085 33791511

[bib35a] Muñoz MM, Losos JB. 2018. Thermoregulatory behavior simultaneously promotes and forestalls evolution in a tropical lizard. Am Nat 191:E15–26. 29244559 10.1086/694779

[bib37] Nakagawa S, Schielzeth H. 2013. A general and simple method for obtaining *R*^2^ from generalized linear mixed-effects models. Methods Ecol Evol 4:133–42.

[bib38] Navas CA, Antoniazzi MM, Carvalho JE, Suzuki H, Jared C. 2007. Physiological basis for diurnal activity in dispersing juvenile *Bufo granulosus* in the Caatinga, a Brazilian semi-arid environment. Compar Biochem Physiol Mol Integrat Physiol 147:647–57. 10.1016/j.cbpa.2006.04.035 17234442

[bib39] Navas CA, Gouveia SF, Solano-Iguarán JJ, Vidal MA, Bacigalupe LD. 2021. Amphibian responses in experimental thermal gradients: concepts and limits for inference. Comp Biochem Physiol B Biochem Mol Biol 254:110576. 33609807 10.1016/j.cbpb.2021.110576

[bib40] Ørsted M, Jørgensen LB, Overgaard J. 2022. Finding the right thermal limit: a framework to reconcile ecological, physiological and methodological aspects of CTmax in ectotherms. J Exp Biol 225:1–15. 10.1242/jeb.244514 36189693

[bib41] Paranjpe DA, Cooper RD, Patten A, Sinervo B. 2012. Measuring thermal profile of reptiles in laboratory and field. Proceedings of Measuring Behavior. 28–31.

[bib3a] Pinheiro J, Bates D. 2000. Mixed-effects models in S and S-PLUS. Springer Science & Business Media.

[bib42] Platt JR . 1964. Strong inference. Science 146:347–53. 17739513 10.1126/science.146.3642.347

[bib43] Pough FH, Gans C. 1982. The vocabulary of reptilian thermoregulation. Biol Reptilia 12:17–23.

[bib44] Reiter MK, Anthony CD, Hickerson C-AM. 2014. Territorial behavior and ecological divergence in a polymorphic salamander. Copeia 2014:481–8.

[bib45] Ribeiro PL, Camacho A, Navas CA. 2012. Considerations for assessing maximum critical temperatures in small ectothermic animals: insights from leaf-cutting ants. PLoS One 7:e32083. 22384147 10.1371/journal.pone.0032083 PMC3286443

[bib45a] Riddell EA, McPhail J, Damm JD, Sears MW. 2018. Trade‐offs between water loss and gas exchange influence habitat suitability of a woodland salamander. Funct Ecol 32:916–25.

[bib46] Stoffel MA, Nakagawa S, Schielzeth H. 2017. rptR: repeatability estimation and variance decomposition by generalized linear mixed-effects models. Methods Ecol Evol 8:1639–44.

[bib47] Sugalski MT, Claussen DL. 1997. Preference for soil moisture, soil pH, and light intensity by the Salamander, *Plethodon cinereus*. J Herpetol 31:245.

[bib48] Test FH . 1946. Relations of the red-backed salamander (*Plethodon cinereus*) to light and contact. Ecology 27:246–54.

[bib49] Touska F, Winter Z, Mueller A, Vlachova V, Larsen J, Zimmermann K. 2016. Comprehensive thermal preference phenotyping in mice using a novel automated circular gradient assay. Temperature 3:77–91. 10.1080/23328940.2015.1135689 PMC4861200 27227099

[bib50] Treit D, Fundytus M. 1988. Thigmotaxis as a test for anxiolytic activity in rats. Pharmacol Biochem Behav 31:959–62. 3252289 10.1016/0091-3057(88)90413-3

[bib51] Vernberg FJ . 1955. Correlation of physiological and behavior indexes of activity in the study of *Plethodon cinereus* (Green) and *Plethodon glutinosus* (Green). American Midland Naturalist 54:382.

[bib52] Waldron BP, Campbell CA, Kuchta SR. 2024. Specialist or refugee: microhabitat use and competition between two sympatric woodland salamanders. J Zool 323:45–56.

[bib53] Waldron BP, Ganzfried MC, Hickerson CAM, Anthony CD. 2022. Repeatability of foraging behavior following a simulated predation attempt depends on color morph, sex, and foraging metric in red-backed salamanders (*Plethodon cinereus*). Ethol Ecol Evol 34:471–84.

[bib45ab] Wu NC, Bovo RP, Enriquez-Urzelai U, Clusella-Trullas S, Kearney MR, Navas CA, Kong JD. 2024. Global exposure risk of frogs to increasing environmental dryness. Nat Clim Change 14:1314–22.

[bib54] Wyman RL . 1988. Soil acidity and moisture and the distribution of amphibians in five forests of Southcentral New York. Copeia 1988:394.

[bib55] Wyman RL, Hawksley-Lescault DS. 1987. Soil acidity affects distribution, behavior, and physiology of the Salamader *Plethodon cinereus*. Ecology 68:1819–27. 29357182 10.2307/1939873

