# Supplementary material for: Revisiting Thermal Gradient Experiments: Effects of Thermal Heterogeneity on Salamander Behavior
Source: Integr Org Biol. 2025 Apr 8;7(1):obaf015. doi: 10.1093/iob/obaf015 (PMC12046510; doi:10.1093/iob/obaf015)
Supplement: obaf015_Supplemental_File [file obaf015_supplemental_file.pdf]

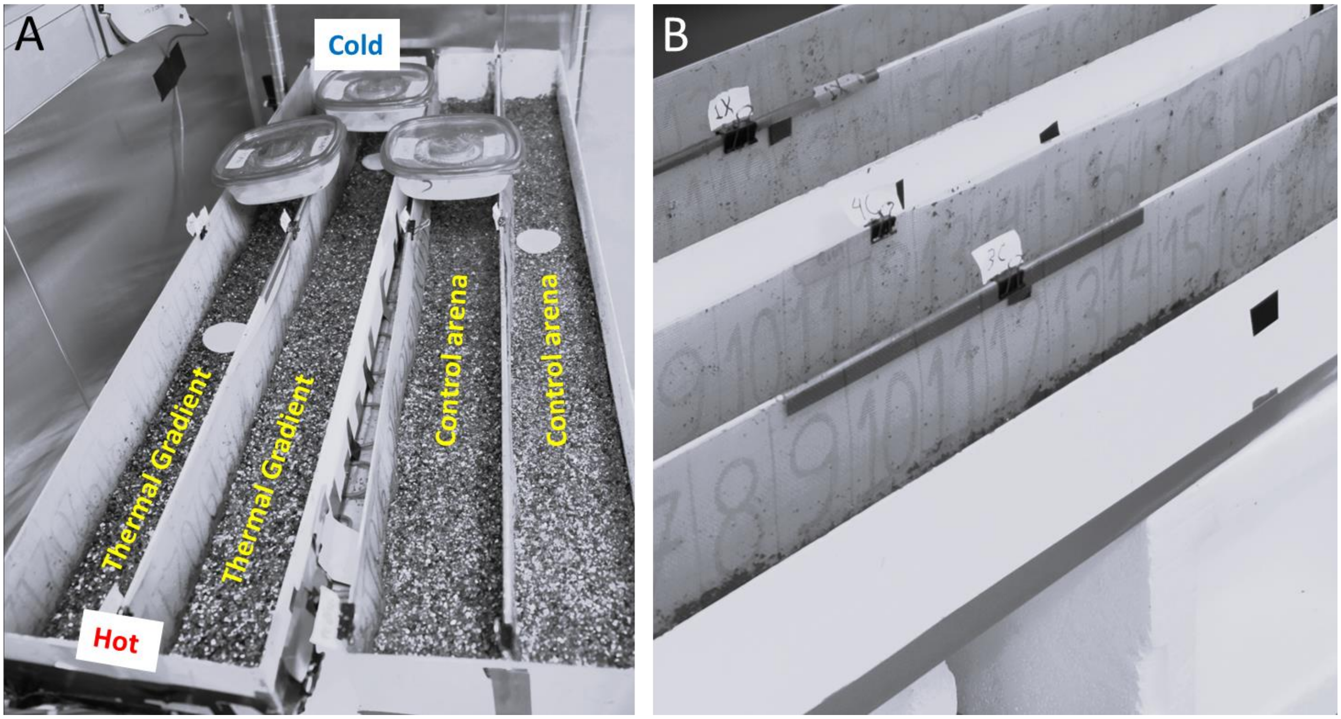

Figure S1. A) Experimental unit composed by two rectangular systems, one used as control (room temperature) and the other used as experimental (gradient). To avoid thermal interference, Gradients and Controls were paired. B) Arenas were divided into 22 segments.

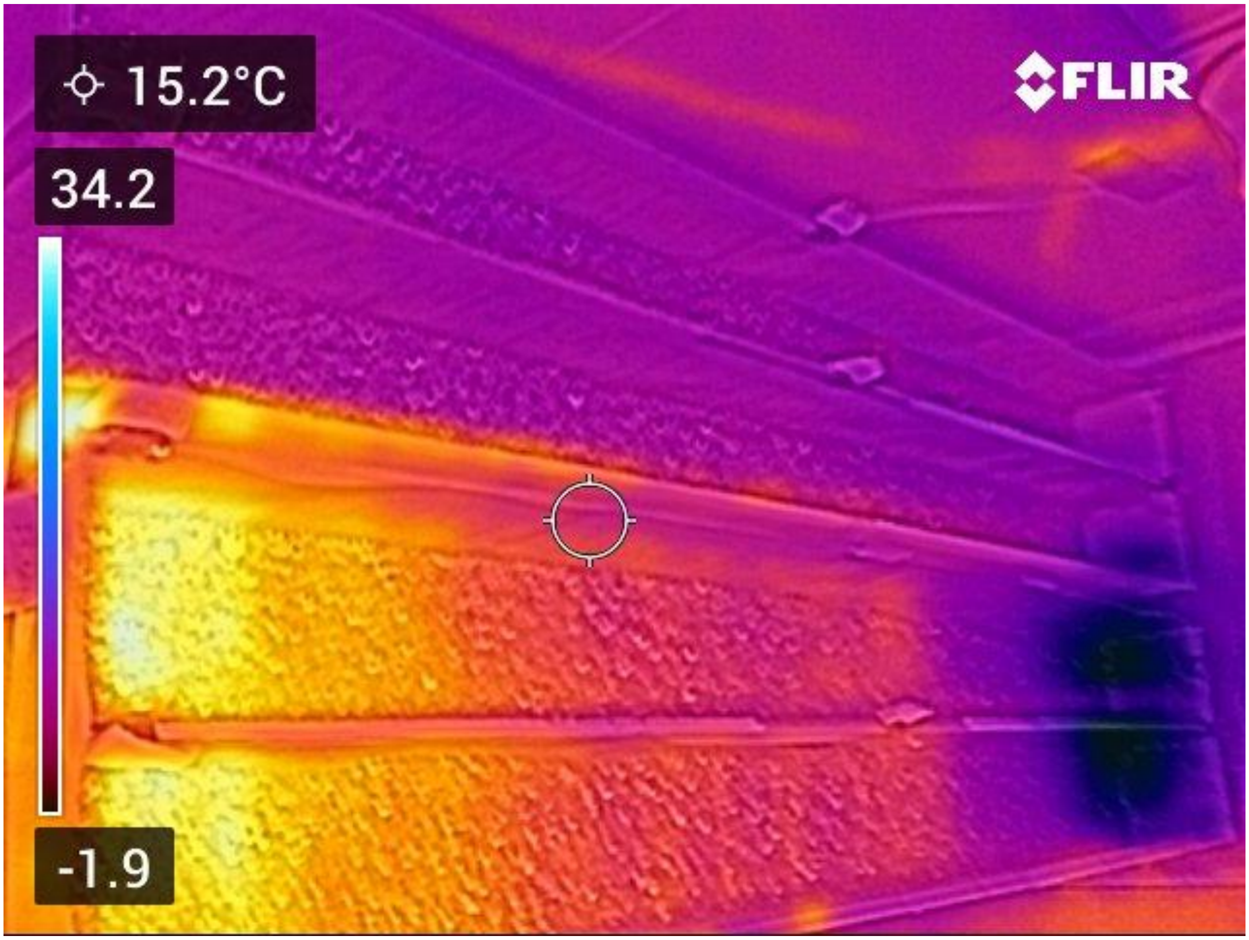

Figure S2. Illustrative thermographic image of the experimental thermal gradient and their contrast with controls.

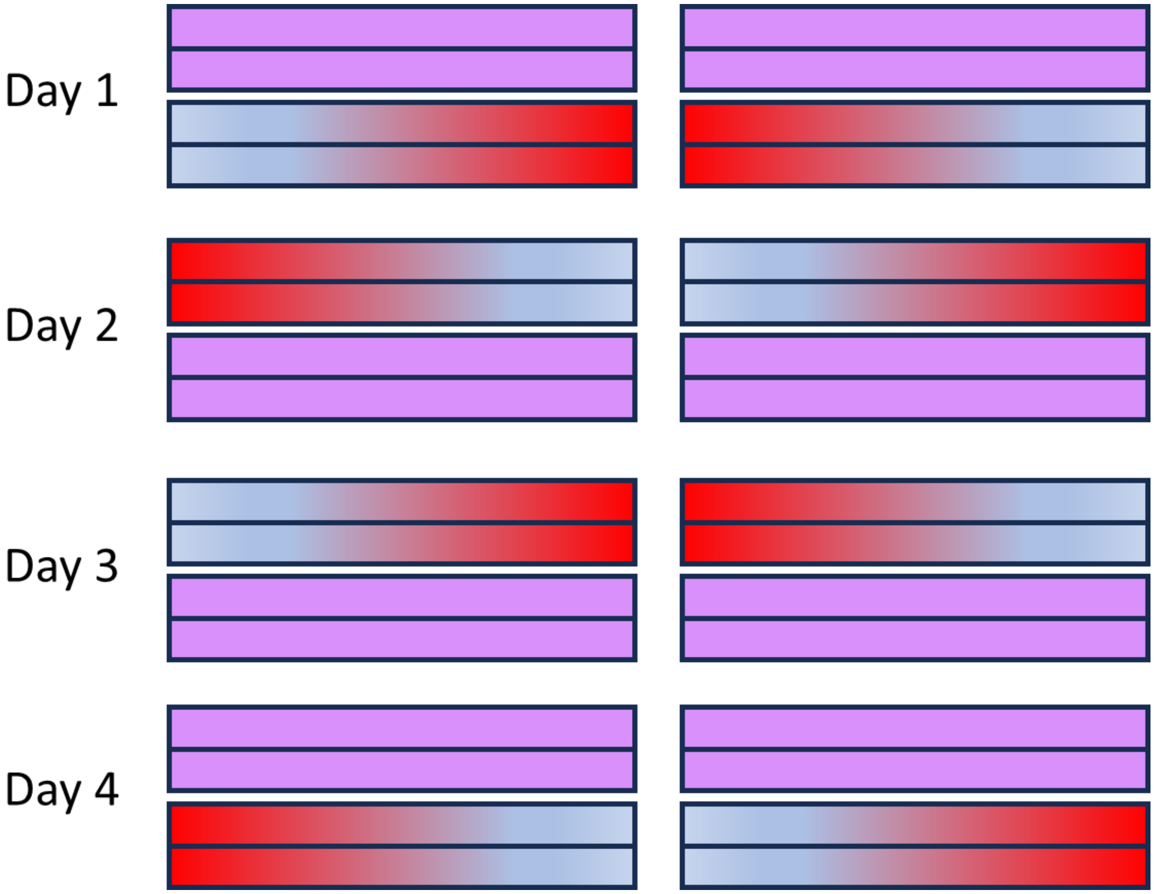

Figure S3. Changes in the experimental routine of variables related to orientation and location of the experimental set up. The table shows the alternatives and their order. This sequence was repeated until the end of experiments. Day refers to experimental day. Day 5 was equal to Day 1, and so on. Controls are represented in purple, and gradients in blue (cold) to red (hot).

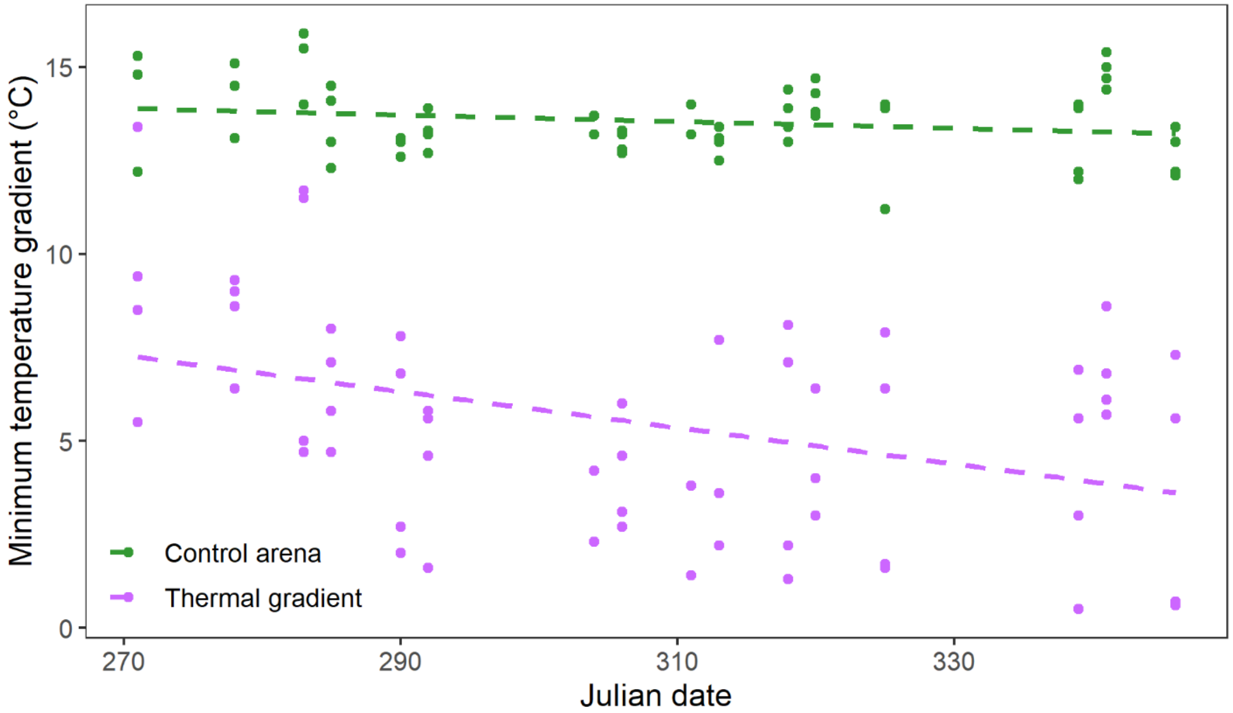

Figure S4. Scatter plot showing minimum temperature in control arenas (green) and thermal gradients (purple) through time (Julian date). Note that in gradients minimum temperatures tended to decrease throughout the season, while control minimum temperatures tended to stay consistent.

Table S1. Ranges (minimum – maximum) for variables measured, in control arenas and thermal gradients. For details on specific variables please refer to the Methods section.

**Variable**	**Unit Description**	**Control**	**Gradient**
Reaches to number 22 (Warmest)	*Count*	0 – 5	0 – 5
Reaches to number 1 (Coldest)	*Count*	0 – 8	0 – 7
Coast to coast events (1 to 22)	*Count*	0 – 8	0 – 8
Mean position in the gradient	*Position number (from 1 – 22)*	1 – 21.91	1.06 – 20.36
Total movement	*Count (segments moved)*	0 – 185	1 – 96
Motionlessness	*Count*	4 – 21	6 – 20
Maximum number visited	*Position number (from 1 – 22)*	1 – 22	2 – 22
Minimum number visited	*Position number (from 1 – 22)*	1 – 20	1 – 17
Time at last movement	*Time (minutes)*	0 – 105	5 – 105
Number of movement events	*Count*	0 – 13	1 – 13
Dorsal Temperature (extrapolated in control)	*Temperature (°C)*	4.1 – 32.8	2.5 – 25.1
